# An Automated Paradigm for *Drosophila* Visual Psychophysics

**DOI:** 10.1371/journal.pone.0021619

**Published:** 2011-06-29

**Authors:** Oliver Evans, Angelique C. Paulk, Bruno van Swinderen

**Affiliations:** Queensland Brain Institute, The University of Queensland, Brisbane, Queensland, Australia; Max-Planck Institute of Neurobiology, Germany

## Abstract

**Background:**

Mutations that cause learning and memory defects in *Drosophila melanogaster* have been found to also compromise visual responsiveness and attention. A better understanding of attention-like defects in such *Drosophila* mutants therefore requires a more detailed characterization of visual responsiveness across a range of visual parameters.

**Methodology/Principal Findings:**

We designed an automated behavioral paradigm for efficiently dissecting visual responsiveness in *Drosophila*. Populations of flies walk through multiplexed serial choice mazes while being exposed to moving visuals displayed on computer monitors, and infra-red fly counters at the end of each maze automatically score the responsiveness of a strain. To test our new design, we performed a detailed comparison between wild-type flies and a learning and memory mutant, *dunce* ^1^. We first confirmed that the learning mutant *dunce* ^1^ displays increased responsiveness to a black/green moving grating compared to wild type in this new design. We then extended this result to explore responses to a wide range of psychophysical parameters for moving gratings (e.g., luminosity, contrast, spatial frequency, velocity) as well as to a different stimulus, moving dots. Finally, we combined these visuals (gratings versus dots) in competition to investigate how *dunce* ^1^ and wild-type flies respond to more complex and conflicting motion effects.

**Conclusions/Significance:**

We found that *dunce* ^1^ responds more strongly than wild type to high contrast and highly structured motion. This effect was found for simple gratings, dots, and combinations of both stimuli presented in competition.

## Introduction

Animals respond reflexively to motion that they see in their environment. This reflex has been termed an optomotor or optokinetic response, depending on whether movement of the whole animal or just the eye is measured, respectively [1], [2], [3]. Such responses have been extensively studied in insects [4], [5], [6], and recent work in the fly *Drosophila melanogaster* has identified key peripheral circuits in the fly visual system believed to be involved in mediating these responses [7], [8], [9], [10]. However, it is likely that visual responses can be modulated or even suppressed by processing occurring in the central brain since, like many animals, flies must be able to ignore certain motion cues while moving through the environment. Investigations of *Drosophila* learning and memory mutants have uncovered a wide range of effects on visual responses in flies [11], [12], [13], [14], [15], [16]. In particular, mutants affecting cyclic AMP signaling, such as the phosphodiesterase mutant *dunce* ^1^, or the adenylyl cyclase mutant *rutabaga^2080^*, were found to display increased visual responsiveness compared to wild type in a choice maze paradigm [11], and this behavior was associated with an attention-like defects in the mutants [13]. A subsequent screen of long-term memory mutants uncovered other strains with increased visual responsiveness in the same paradigm, and these were also associated with attention-like defects at the level of both behavior and electrophysiology [15].

A systematic analysis of visual psychophysics in a learning and memory mutant such as *dunce* ^1^ has never been done. In part, this is because there have been few paradigms available to efficiently test a variety of visual scenarios in fly populations, and also because mutants such as *dunce* ^1^, which do not fly readily, are difficult to investigate thoroughly in the best visual paradigm to date, the tethered flight arena [5]. We have therefore applied our automated visual maze design to better characterize vision in *dunce* ^1^ flies compared to wild type. We questioned whether increased visual responsiveness of the *dunce* mutant in our paradigm was due to improved visual processing in general or increased responsiveness to a narrow range of physical parameters. We addressed this problem by testing *dunce* ^1^ and wild-type flies to a wide range of moving visual stimuli, including different gratings, moving dots, or more complex visual stimuli. Our comparative psychophysical study of *dunce* ^1^ against wild type shows that *dunce* ^1^ flies respond strongly to highly structured motion stimuli, whether these are gratings or dots. The tight association between a learning mutant and a stronger visual response across different physical parameters suggests that our automated paradigm will be useful for efficiently screening other genes involved in plasticity mechanisms.

## Results

### Visual Responses

**Figure 1:**
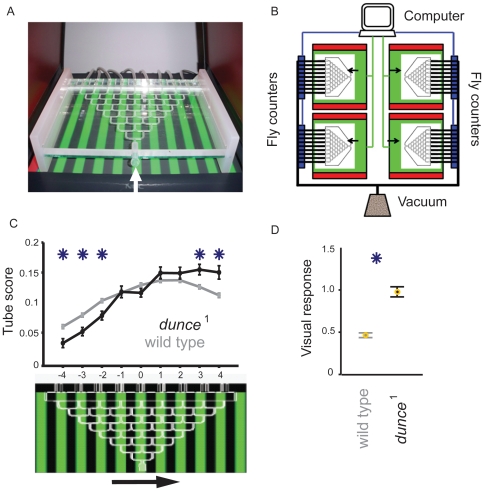
The maze paradigm. **A.** A maze over a CRT displaying a grating stimulus. After completing the maze, flies (∼30 per maze) are vacuumed from the nine collection chambers and automatically counted. Arrow: entry into the maze. **B.** Schematic of automated setup. Green boxes are CRTs, red rectangles are side LCDs, black lines represent the vacuum system sucking flies through infra-red counters (blue rectangles) following an experiment, to be disposed of in a morgue. Data from multiple mazes are averaged to calculate visual responsiveness for a strain or condition. **C.** Flies follow the direction of motion displayed on the CRT monitor (lower panel, grating is moving right), which results in a larger number of flies counted in tubes 1 to 4 versus those in tubes −1 to −4. *dunce* ^1^ flies (shown in black, ± s.e.m, N = 50 mazes of ∼30 female flies each) respond significantly more strongly to a moving green/black grating than wild type (in gray, ± s.e.m, N = 488 mazes of ∼30 female flies each). *, significantly different proportion in tube (*P*<0.05, by *t*-test). **D.** Visual Responsiveness (VR) is calculated as the weighted average of fly distribution in the maze. VR averages (± s.e.m) are shown for wild type and *dunce* ^1^ (*, *P*<0.01, by *t*-test).

We used an automated paradigm to measure visual responsiveness in walking *Drosophila* (Figure 1A,B, and see Materials and Methods). In this set-up, *dunce* ^1^ flies display increased visual responsiveness to moving gratings, compared to wild type [13], [15]; this result was replicated with a good level of reliability in our high-throughput design (Figure 1 C,D; N = 50 mazes for *dunce* ^1^ and 488 mazes for wild type, with about 25 flies per maze). Our design allowed us to then efficiently test other visual stimuli, probing for example whether *dunce* ^1^ has improved visual acuity compared to wild type. We tested this possibility by manipulating the visual stimulus across a range of physical parameters and motion complexity.

**Figure 2:**
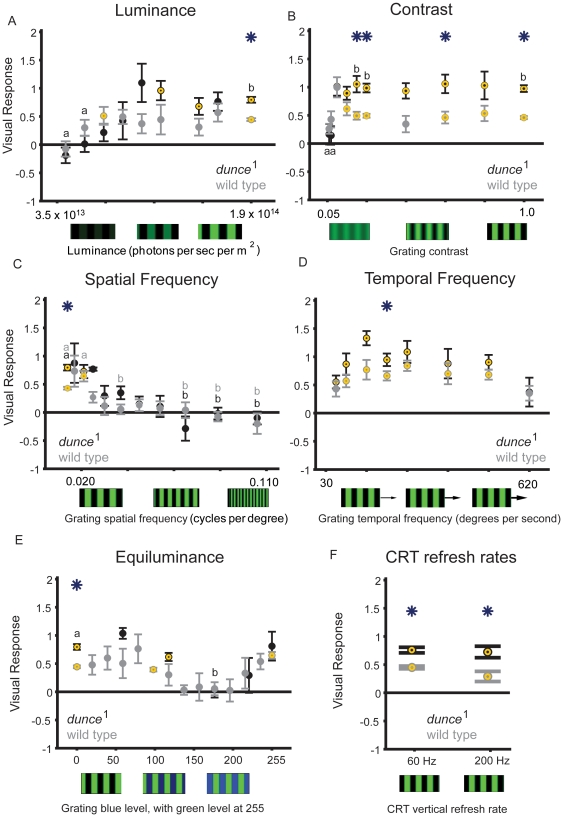
Changing grating parameters. **A.** Wild type (gray) and *dunce* ^1^ (black) responses to changes in green luminance (all other grating parameters are standard, as described in the Metods). **B.** Wild type and *dunce* ^1^ flies responsivenss to changes in contrast. **C.** As the spatial frequency increased (velocity maintained constant), *dunce* ^1^ and wild-type flies decreased their response to the moving gratings. **D.** The grating velocity profile is similar between *dunce* ^1^ and wild type, with decreased responsiveness for bars moving very fast or very slowly. **E.** Equiluminance experiments. The luminance of a moving blue grating on a constant green background was gradually increased in different experiments. Visual responsiveness for both *dunce* ^1^ and wild type is lost when blue and green are equiluminous (∼175 blue versus 255 green intensity, see Methods). The same experiment was also performed with changing green luminance on a constant blue background, with qualitatively similar results (not shown). **F.** Screen refresh rates. We exposed *dunce* ^1^ and wild-type flies to the standard green/black moving grating (as in Figure 1) at different refresh rates for the CRT computer monitor (see Methods). Above the presumed flicker fusion frequency for fly vision (∼200 Hz [5]), visual responses were not different than for our standard 60 Hz refresh, with *dunce* ^1^ respondes significantly greater than wild type to gratings refreshed at 200 Hz as well as 60 Hz. For all of these experiments (A–F), yellow circles identify significant responses compared to zero, asterisks identify significant differences between the strains (*P*<0.01, by *t*-test), and “a” and “b” identify significantly different groups within a strain (*P*<0.01, by ANOVA, multiple comparisons test; gray, wild type; black *dunce* ^1^).These statistics show similarities between the curves for either strain, also analyzed by correlation statistics in Table 1.

We first investigated whether increased visual responsiveness in *dunce* ^1^ generalizes across different stimulus parameters, or whether it is specific to the one grating stimulus tested above (see Materials and Methods). We found that, with the exception of changes in grating contrast, *dunce* ^1^ mutants were generally not significantly different from wild type when we changed grating luminosity, spatial frequency, and temporal frequency (Figure 2A–D). Indeed, *dunce* ^1^ visual responsiveness decreased to zero at the same physical settings that abolished responsiveness in wild type, such as low luminance, low contrast, and high spatial and temporal frequencies (see Table 1 for correlation statistics between the strains). This suggests that the increased responsiveness in the mutant for our standard stimulus (e.g., Figure 1C, and see Methods) is not due to improved visual acuity, but is instead an increased response under “optimal” visual condition, such as high contrast and luminosity. Furthermore, we observed in both strains a predictable loss of visual responsiveness for gratings with equiluminant green and blue alternating bars (Figure 2E), resulting in a high correlation between the two strains (0.92; p<0.05; Table 1). Loss of optomotor responsiveness at color equiluminance has been reported previously as evidence for segregation of color and motion processing in flies in a tethered flight paradigm [17]. Finally, the increased responsiveness of *dunce* ^1^ was maintained for higher monitor refresh rates (200 vs 60 Hz, Figure 2F). Together, these data suggest that *dunce* ^1^ visual responses are unlikely to stem from peripheral visual processing stages likely to affect detecting luminance, low contrast, spatial frequency, flicker, or velocity computations. In addition, our results validate the usefulness of the maze paradigm to investigate fly vision. Although the maze paradigm is a population assay comprising various behaviors other than classical optomotor responses, it produces visual responses in accord with expectations for the optomotor conditions explored by researchers in the past walking or flight paradigms. For example, flies in the maze display syndirectional responses to moving gratings (Figure 1C) [5], [18], responses are maximal under high luminosity and high contrast (Figures 2A & 2B) [5], [19], flies lose responsiveness at high spatial frequencies (Figure 2C) and flies display a velocity response curve (Figure 2D) [4], [5], and flies lose responsiveness under color equiluminance (Figure 2E) [17]. These results encouraged us to proceed to explore responses to other, more complex visual stimuli in the maze paradigm.

**Table 1:** Correlation statistics between *dunce* ^1^ and visual parameter (Stimulus, in referenced Figure panel number), wild type and visual parameter, and *dunce* ^1^ versus wild type.

	*dunce* ^1^: correlation to visual stimuli		Wild type: correlation to visual stimuli		Correlation of wild-type and *dunce* ^1^ visual responses	
Stimulus	r value	p-value	r value	p-value	r value	p-value
Figure 2 A	**0.466**	**0.000**	0.065	0.221	**0.591**	**0.094**
Figure 2 B	**0.138**	**0.046**	−0.038	0.356	0.441	0.203
Figure 2 C	**−0.512**	**0.000**	**−0.296**	**0.000**	**0.851**	**0.001**
Figure 2 D	−0.122	0.234	0.016	0.795	**0.790**	**0.020**
Figure 2 E	**−0.328**	**0.001**	−0.078	0.133	**0.920**	**0.009**
Figure 4 A	**0.586**	**0.001**	0.122	0.124	0.508	0.661
Figure 4 B	−0.127	0.315	−0.133	0.086	**0.694**	**0.038**
Figure 4 C	**0.460**	**0.000**	**0.184**	**0.034**	0.274	0.553
Figure 4 D	0.157	0.130	**0.369**	**0.000**	0.310	0.303
Figure 4 E	**−0.372**	**0.022**	**−0.300**	**0.012**	0.686	0.201
Figure 4 F	0.202	0.067	**0.305**	**0.001**	0.498	0.143
Figure 6 A	−0.042	0.636	0.118	0.119	−0.210	0.560
Figure 6 B	**−0.632**	**0.000**	**−0.280**	**0.000**	**0.793**	**0.001**

Significant correlations (*P*<0.05) are indicated in bold type.

**Figure 3:**
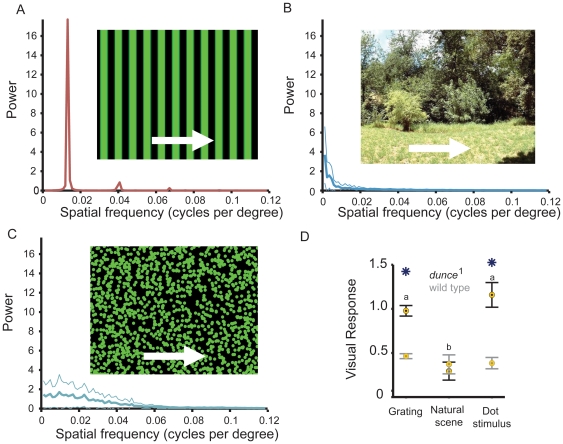
Responses of wild type and *dunce* ^1^ to more complex wide-field stimuli. **A.** The grating stimulus and associated spectral analysis of the image used in Figure 1 (See Methods). **B.** A natural scene moving at 130 degrees/s, the same velocity as the grating in Figure 1. A power spectrum (± s.e.m.) for the image is shown. **C.** A random dot stimulus (1500 green coherent dots of 13.8 degrees visual subtense width, 10 sec lifespan, moving at 130 degrees/s; a power spectrum (± s.e.m. for the image is shown (see http://web.qbi.uq.edu.au/vanswinderen/Movie2.mpeg for the stimulus). **D.** Visual responsiveness to either stimulus for wild type (gray) and *dunce* ^1^ (black). Yellow circles identify significant responses compared to zero, asterisks identify significant differences between the strains (*P*<0.05, by *t*-test), and “a” and “b” identify significantly different groups within a strain (*P*<0.01, by ANOVA, multiple comparisons test).

Visual responses explored thus far have been to moving gratings, a stimulus with straight edges and a high level of regularity (as exemplified by the single peaks in a spectral analysis of the image, Figure 3A) that evokes strong visual reflexes in flies and other insects [4], [5], [20]. One possible explanation for *dunce* ^1^ behavior in the visual maze is that the mutant is more responsive to moving straight edges. We therefore tested responses to a natural scene (Figure 3B) and to random dots (Figure 3C), two stimuli that include a variety of moving edges with a range of spatial frequencies (see Materials and Methods). Random dot kinematograms (RDK) have been used extensively in visual studies in humans and monkeys for investigating how local motion is integrated into global motion [21], [22], [23], and for studies of visual attention in humans [24]. RDKs also reveal responses to irregular wide-field motion more typical of natural scenes, and thereby provide a more flexible stimulus in terms of the number and types of parameters that can be manipulated compared to gratings.

The separation between *dunce* ^1^ and wild type was lost when we tested responsiveness to a natural scene (Australian bushes [25]), but was maintained with a green random dot stimulus (Figure 3D, and see Materials and Methods for image parameters). Given the high energy of the natural scene at low spatial frequencies (see spectrogram inset in Figure 3B), this result may be surprising. However, we showed earlier that *dunce* ^1^ responsiveness was reduced to wild-type levels for most luminance levels (Figure 2A) and spatial frequencies (Figure 2C) beyond our standard grating (see Methods), suggesting that *dunce* ^1^ increased responsiveness to the grating operates within a narrow range of physical parameters not captured by the natural scene we tested. It was therefore surprising that the random dot stimulus, which more resembles the natural scene spectrally (Figure 3C), resurrected the *dunce* ^1^ phenotype. We therefore focused on RDKs to better explore the physical parameters of this alternate stimulus that might be evoking a stronger response in *dunce* ^1^.

**Figure 4:**
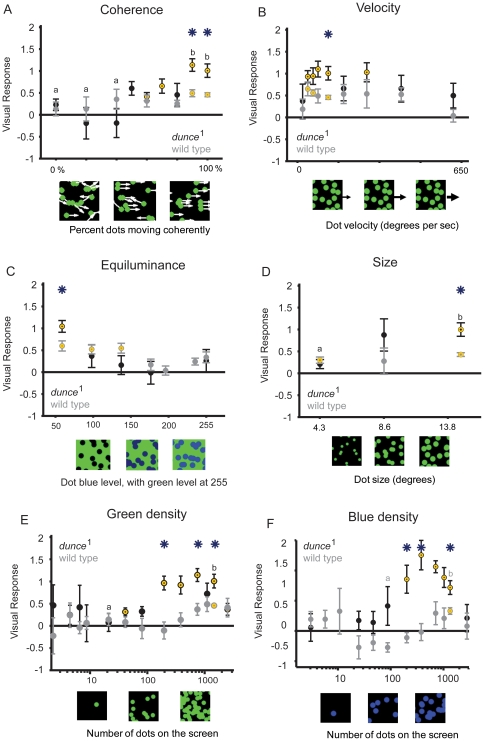
Random dot responses. **A.** Increasing dot motion coherence from 0% to 100% resulted in significant differences between the strains *dunce* ^1^ (black) and wild-type (gray) only once 80% of dots were moving coherently. All other parameters are standard (see Methods) **B.** *dunce* ^1^ and wild type displayed decreased visual responsiveness for slow or fast-moving dots, while the strains were significantly different at intermediate velocities. **C.** Both strains displayed similar equiluminance curves, where blue intensity was increased against a standard green set at 255. **D.** *dunce* ^1^ and wild-type responsiveness to different dot sizes. Degrees are subtended relative to a fly in the maze looking at the CRT screen below (see Methods). **E.** *dunce* ^1^ was more sensitive to lower densities of green dots than wild type (See Methods for other parameters kept constant). The x axis is log scale. **F.** The separation between *dunce* ^1^ and wild-type was also evident for increasing blue dot densities. The x axis is log scale. In all graphs, asterisks indicate when strain values are significantly different from one another, *P*<0.01, yellow circles indicate that the visual responses are significantly different from zero, *P*<0.01, “a” and “b” indicate statistically different groups within a strain (*P*<0.01, by ANOVA, multiple comparisons test *dunce* ^1^ is black, wild type is gray).

As for gratings, *dunce* ^1^ responsiveness to moving dots was stronger than wild type only for a narrow range of physical parameters, and responses in both strains were mostly well correlated (Figure 4, and Table 1). Both strains for example required a similar level of coherent motion to evoke a response (about 80% motion coherence), both were similarly affected by changes in dot velocity (losing responsiveness at the same high and low velocities), both lost responsiveness when dots were equiluminant to the background, and *dunce* ^1^ was also not more sensitive to smaller dots (Figure 4A–D, and see Table 1). Where we did find a significant difference and lack of correlation between the strains was in response to dot densities: *dunce* ^1^ responded strongly to intermediate densities, where wild type showed no significant response (Figure 4E). This effect was true for blue dots as well (Figure 4F). Interestingly, wild-type flies displayed a tendency toward a negative response for sparse blue dot densities, which is consistent with another study examining fly responses to sparse moving dots [8]. Positive responses to fewer moving dots in *dunce* ^1^ suggests a decreased arousal threshold – or increased sensitivity – to wide-field motion in the mutant.

### Visual competition

**Figure 5:**
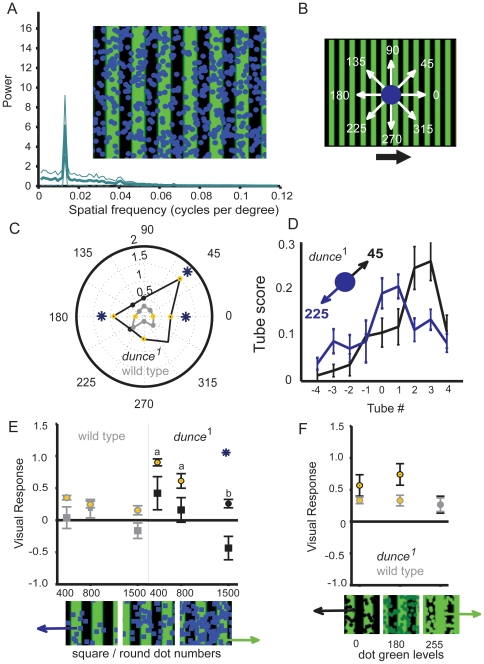
Combining the wide-field stimuli. **A.** ∼500 coherent blue dots were layered onto the moving grating (see http://web.qbi.uq.edu.au/vanswinderen/Movie3.mpeg). A power spectrum (± s.e.m.) for the image is shown (see Methods). **B.** The dot stimulus was moved in eight different directions relative to the grating motion, with 0° representing coherent movement with the grating and 180° movement against the grating; relative grating direction is indicated by the black arrow; the maze is placed over the grating as in Figure 1C. **C.** Polar plot of visual responses for both strains responding to the combined stimuli for 8 different dot motion directions (gray: wild type; black: *dunce* ^1^). *, significantly different between the stains; yellow dot, significantly different from zero, *P*<0.01. **D.** Distribution of *dunce* ^1^ flies in the maze (proportion in tube ± s.e.m.) for two different superimposed dot orientations (45° and 225°). **E.** The effect of changing dot shape from circles to squares (moving at 180° against the grating), as indicated by the shape of the points. *, significantly different response between circles or squares, *P*<0.01. **F.** Changing dot luminosity. In the two extreme conditions (green = 0 and green = 255), dots are either fully black or green. For all VR data, *  =  significantly different response between wild type (gray) and *dunce* ^1^, *P*<0.01; yellow circles indicate that the visual responses are significantly different from zero, *P*<0.01.

To better understand how flies might be integrating motion cues, we combined our dot and grating stimuli (Figure 5A), thereby asking: how do wild-type and mutant flies respond behaviorally to the stimuli presented in competition? A human observer can easily attend to one or the other stimulus separately (see http://web.qbi.uq.edu.au/vanswinderen/Movie3.mpeg), but what about flies? One possibility is that flies might respond - like humans paying attention - alternately to two competing percepts (e.g., wide-field gratings versus dots); another possibility is that the combined visuals present a degraded motion percept to the flies. Both possibilities could produce a zero response score on our paradigm, and to separate these two possibilities behaviorally is difficult. However, electrophysiological recordings in insects have identified neurons that respond specifically to small moving targets, such as dots [26], [27], and other neurons that respond specifically to wide-field motion [28], [29], so in principle it is conceivable that either system might be modulated separately to affect behavioral choices.

We first tested whether superimposed moving dots could alter the response to the moving grating. A set number of blue dots (∼500) were moved coherently over the standard grating in eight different relative directions (Figure 5B). We found that superimposed dots evoked different responses in wild type and *dunce* ^1^ depending on their motion direction relative to the grating (Figure 5C). Notably, *dunce* ^1^ responsiveness was increased by one superimposed orientation (45°, VR = 1.52±0.172) and correspondingly decreased by the opposite orientation (225°, VR = 0.52±0.134, Figure 5D). This suggests that the combined stimuli may be acting additively for *dunce* ^1^, while effects on wild type are not additive or not as salient.

To further probe additive effects of the combined stimuli, we changed dot shape or luminosity over the grating stimulus. When square dots were presented instead of round (at 180°, or against the grating direction), both strains lost responsiveness to the grating, and this effect was repeated for different dot densities (Figure 5E). It is impossible for this result to be explained by the dots simply subtracting grating surface area since the number of pixels per square or circle was the same; rather, square dots provide a more salient competing stimulus than round dots. We also changed the luminosity of superimposed green dots on the green grating, and found that differences between wild type and *dunce* ^1^ were lost when the competing stimuli were equiluminant (Figure 5F). Together, these data show that *dunce* ^1^ is especially sensitive physical aspects of the superimposed dot stimulus, even though the grating surface covered by the dots may remain unchanged. This does not appear to be the case for wild type, which is less responsive to the superimposed stimulus, as it is less responsive to the grating alone.

**Figure 6:**
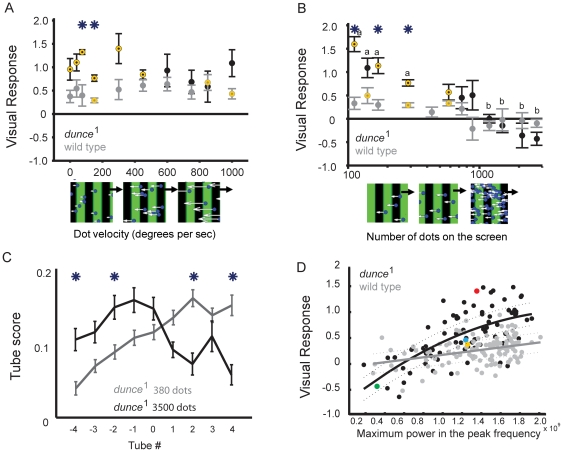
Titrating wide-field competition effects. **A.** Increasing the velocity of ∼500 coherent blue dots moving in opposite direction (180°) to the standard grating produced opposite effects in *dunce* ^1^ and wild-type flies, causing differences between the strains to be lost at intermediate dot velocities. Asterisks indicate when *dunce* ^1^ (black) and wild-type (gray) values are significantly different from one another, *P*<0.01. Yellow indicates that the visual responses are significantly different from zero, *P*<0.01. **B.** Increasing dot density (shown log scale) abolished responsiveness to the grating in both strains. Blue dots were presented flowing coherently in opposite direction to the moving grating (180°), at a set velocity (130 degrees per sec). Flies displaying a positive Visual Response are moving in the direction of the grating (wild type, gray; *dunce* ^1^, black). **C.** *dunce* ^1^ distribution in the maze (proportion in tube ± s.e.m.) for 380 vs 3500 blue dots superimposed on the grating. **D.** Meta analysis for all experiments comparing *dunce* ^1^ (black) to wild type (gray) with combined dot and grating visuals, plotting Visual Response against the power of the peak frequency in all movies where dots and gratings were combined (see Methods). The strains respond similarly to images with low to intermediate motion regularity (power of the peak frequency), but diverge when image motion is more structured (high power). Data for each strain have been fit by a polynomial function for *dunce* ^1^ (y = -633.5x^2^+71.3x+−0.8940, correlation  = 0.56, *P*<0.05) and a linear fit for wild type (y = 8.7297x+−0.0347, correlation  = 0.14, *P*<0.05), where y is the visual response and x is the power of the peak frequency. Fits are shown ± s.e.m. Select *dunce* ^1^ experiments are superimposed in color: yellow-green: square versus round dots (Figure 5E); red-blue: 45° versus 225° orientation (Figure 5C).

Having changed the orientation, shape, and luminance of the superimposed dots, we next assessed the effect of their velocity and number on the grating response. Increasing the velocity of the (∼500) competing blue dots had no significant effect on wild-type responsiveness to the grating; responsiveness even increased slightly (Figure 6A, gray circles). In contrast, *dunce* mutants exposed to the same scenario showed a generally degraded visual response with increasing dot velocity (Figure 6A, black circles). Notably, at high dot velocities (>400 pixels/s), responsiveness levels between *dunce* ^1^ and wild type were not significantly different from each other. Interestingly, a resurrection of *dunce* ^1^ responsiveness to the grating was noted at highest dot velocities (1000 pixels/s, as seen in Figure 6A). This would be expected because the competing dots probably lose motion coherence at high velocities (because they would we “skipping” incoherently when displayed at a 60 Hz refresh). The behavior of *dunce* ^1^ in this last experiment supports the possibility that dots are acting as a competing percept, rather than merely subtracting from the grating response.

As we have seen, *dunce* ^1^ displayed increased responsiveness to fewer moving dots than wild type (Figure 4E&F). To better understand how either strain might be responding to the combined wide-field stimuli, we presented increasing numbers of blue dots moving coherently against the direction of motion of the grating. We found that above the threshold when *dunce* ^1^ responds to dots alone (∼200 dots), *dunce* ^1^ responsiveness decreased linearly with increasing dot number over the grating (r = −0.62), whereas, in wild-type flies, the same experiment revealed a lower correlation to dot number (r = −0.28, Figure 6B). Although the response profile for either strain is different, responsiveness to the grating is lost at similar dot numbers for wild type and the mutant. *dunce* ^1^ responsiveness shifted significantly toward negative values (in favor of dots) at higher densities of the competing stimulus, as shown in a plot of the distribution of flies in the maze end-tubes (Figure 6C). The linear response of *dunce* ^1^ to increasing numbers of competing dots is consistent with effects due to dot orientation we uncovered in Figure 5C, suggesting additive (or subtractive) effects on the motion pathway.

In our experiments, we have so far assumed what the flies perceive based on a number of defined visual parameters (e.g. grating resolution, dot number). Another way of addressing differences in visual perception between mutants and wild type would be to quantify a common metric across all of the different visuals. One interpretation based on our results is that *dunce* mutants could for example be responding more to wide-field image regularity, regardless of whether these are dots or gratings or combined stimuli. In this view, *dunce* ^1^ visual responsiveness to the combined stimuli would be less tied to competing motion effects, and more tied to overall image regularity. One way of quantifying image regularity is by measuring the power of the dominant spatial frequency in the visuals (See Methods). Exploiting the data-mining capacities of our automated system (all data are appended to a Matlab structure), we analyzed all of the movies used in this study and plotted the maximal spatial frequency power against all visual responses for every experiment where dots and grating were combined (1067 maze runs). The result of our meta-analysis suggests that *dunce* ^1^ are indeed responding strongly to increased image regularity: the stronger the dominant frequency in the visual, the stronger is the mutant's response (Figure 6D). This approach to analyzing our data explains some observations quite well, for instance the stronger effect of moving square dots compared to round dots over a grating (from Figure 5E, shown here for *dunce* ^1^ in yellow and green, respectively). However, this approach does not adequately explain why in other instances, even when the dominant frequency is similar, visual responses in *dunce* ^1^ can be significantly different (e.g., dots moving in opposite directions over a grating, from Figures 5C; corresponding data points are highlighted in Figure 6D for 45° in red and 225° in blue).

## Discussion

In this study, we have applied a level of visual investigation routinely used in human visual perception studies to *Drosophila* populations. We achieved this using an automated visual testing paradigm for flies, which combines computer-generated visual displays with simple serial choice mazes and commercially available *Drosophila* counting devices. As a first test, we applied our device to better understanding visual behavior in a classical olfactory learning mutant, *dunce* ^1^. Our results confirm that *dunce* ^1^ affects visual responsiveness, and that visual processing is likely disturbed by the developmental genetic manipulation. The *dunce* ^1^ mutant responds more strongly than wild type to highly structured motion, especially under conditions of high contrast and luminosity. Furthermore, *dunce* ^1^ also responds more strongly to another wide-field motion stimulus, moving dots, and fewer moving dots were required to evoke a positive visual response in the mutant than wild type. Comparisons with wild type suggested two distinct possibilities: either *dunce* ^1^ is responding more strongly due to *improved* visual processing, or arousal thresholds to wide-field motion have been altered in the mutant. The former would involve peripheral systems, whereas the latter might involve central processing in the brain. Our results suggest that visual responses are not generally improved in the mutant, arguing for a more central arousal threshold defect in *dunce* ^1^. Our experiments show that *dunce* ^1^ is highly responsive to wide field motion even across a variety of more complex scenes comprised of overlapping dots and gratings. In general, responsiveness levels in the mutant can be predicted by image regularity (e.g. the amplitude of the dominant frequency (Figure 6D), but our paradigm also opens up the possibility to screen for responses to competing motion stimuli (as in Figure 5).

Insect visual responses have been originally described as hard-wired reflexes, and most studies have therefore logically focused on visual processing in the eye to explain these behaviors. Yet, why (olfactory) learning mutants such as *dunce* ^1^ exert such strong effects on elementary visual behaviors requires some explanation. We propose that processing in the central brain, such as the *dunce* ^1^ effects explored here, may set a responsiveness threshold for motion signals from the periphery, to guide the visual choices made by flies. This view of visual behavior in flies, where central neural processing also drives the behavior, is not necessarily in conflict with the classical “bottom-up” view, which has been aimed at dissecting visual behavior from the periphery. Recent work has shown that central neurons modulate optomotor responses for flight behavior [30]. A necessary next step in *Drosophila* visual studies will be to map the interface between central processes, such as those overlapping with memory systems [15], and the front-end of fly vision.

One explanation for increased visual responsiveness in *dunce* ^1^ may be that the mutant is less able to suppress responses to salient wide-field motion stimuli. In this perhaps counter-intuitive view, increased visual responsiveness in *dunce* ^1^ would represent a *failure* to suppress a salient visual stimulus. Failed suppression mechanisms as an explanation for improved performance would be consistent with the attention-like defects uncovered for *dunce* ^1^ in electrophysiology paradigms [13], [15]. Humans are able to suppress optokinetic reflexes by directing their attention to a visual target surrounded by wide-field motion, but this ability can degrade with age or cognitive dysfunction [31], [32]. Whether similar suppression mechanisms occur in flies is debatable, although some form of stimulus suppression is evident in all fly attention experiments conducted to date [11], [13], [15], [16], [33], [34]. A view centered on attention-like behavior therefore suggests that visual responses in wild-type flies are shaped to some extent by suppression mechanisms.

The counterargument for this suppression hypothesis suggests that *dunce* mutants simply have increased responsiveness to moving objects, without actually being defective in suppressing motion cues. Although this may be possible, we do not believe this to be true for the following reasons. First, *dunce* ^1^ does not respond more strongly than wild type across all stimulus conditions; rather, *dunce* ^1^ responsiveness peaks under rather narrow optimal conditions that for the most part coincide with wild-type peak responsiveness levels. Second, our RDK experiments showed that *dunce* ^1^ responsiveness depends on the visual context: although *dunce* ^1^ responds strongly to fewer moving dots than wild type, the mutant does not respond to a lower percentage of coherently moving dots (Figure 4A). This shows that the increased responsiveness of *dunce* ^1^ to fewer moving dots is abolished when incoherent moving distracters are present (such as a few dots moving in random directions relative to the overall direction of motion), suggesting detection of wide-field motion is not improved in the mutant. Further, in a previous study we found that producing a transient defect in the fly brain by silencing the output of central neurons also increased responsiveness in transgenic flies to levels as high as observed for *dunce* mutants [15]. A failure in brain function, as induced by such transient synaptic silencing experiments, should not be associated with improved performance, unless that function was involved with attenuating performance. Together, this genetic and behavioral evidence makes an interesting prediction: other manipulations that may compromise brain function, such as aging, neurodegeneration, or stress, should also *increase* visual responsiveness in wild-type flies in our paradigm. Such increase in performance as a signature of *failure* would provide a powerful screen in studies aimed at uncovering attention-related mechanisms in *Drosophila*.

## Materials and Methods

### Animals and Preparation

Our wild-type strain was from the Canton-S genetic background (sourced from the Neurosciences Institute (NSI), San Diego), and this specific background was introgressed 5–6 generations into the *dunce* ^1^ mutant that was used for all of our experiments. Flies were raised on standard *Drosophila* yeast-based media and kept on 12∶12 hr light-dark cycle. Adult females (2–7 days old) were collected under CO_2_ anesthesia and put in batches of ∼25 in “jumbo” plastic transfer pipettes (Fisher Scientific) and supplied with a drop of water but no food. Flies were kept overnight in cycle-matched light-dark incubators set at room temperature (22°C) and were tested the next morning, about 16–20 hrs after collection.

### Mazes

A version of the maze paradigm has been reported previously [11]. Earlier designs to fractionate fly populations according to optomotor responses relied on physically rotating drums and manual scoring [2], [18], whereas our design makes use of computer-generated images and automated analysis. The plexiglas 8-point choice mazes are placed over upturned computer monitors on which moving images can be displayed. Turns made by the flies as they walk through the mazes (see [11], [13] for a description of individual fly behavior in the maze, and http://web.qbi.uq.edu.au/vanswinderen/Movie1.avi for a sample experiment) determine their visual response, which is calculated from their distribution among nine collection points at the end of each maze (See Figure 1). Although it is likely that the assay combines a variety of behaviors in addition to visual motion responses (e.g., following or reversing) we did not detect a significant effect of population size on the visual response in female flies [11]. Experiments performed on runs of single flies yielded similar levels of responsiveness as observed in large groups of female flies (data not shown).

The mazes are flanked to the left and right by angled (70°) liquid crystal display (LCD) screens on which images can also be displayed, although for this study the flanking screens always displayed a uniform red background to illuminate the maze for visual inspection (Figure 1A,B). We modified our maze design in order to allow for a high-throughput, automated assessment of visual behavior in a strain. Multiple identical mazes were constructed based on a standard modular design (J&M Specialty Parts, San Diego, California), and these mazes were run in parallel. Upon completion of an experiment (∼2 minutes), flies were trapped in any of nine holding chambers and then cleared by vacuum suction through attached Tygon tubing (Fisher Scientific). The number of flies in each chamber was counted using infrared sensors (modified “Hi-Speed” *Drosophila* Activity Monitors, Trikinetics, Waltham, Massachusetts) placed along the vacuum route; all flies were disposed into a collection trap after being automatically counted (Figure 1B). Any flies remaining in the maze were forced through by air pressure afterwards and excluded from the final tally. All maze experiments were balanced for image direction, with an equivalent number of flies within a strain tested for responses to images moving in the opposite direction on the computer monitor. For this study, each experiment comprised four mazes (with ∼25 flies each) in either direction, thus ∼200 female flies per data point, unless stated otherwise. Upon completion of an experiment (<5 min for both directions), the visual response was automatically calculated, distributions plotted, significance tested, and data saved using custom Matlab (Mathworks) software, and the next experiment could immediately follow. To generate the data for this paper, one individual (OE) loaded over 80,000 flies into about 2500 maze runs.

### Visual Stimuli

All visual stimuli were made in Vision Egg [25] using Python programming language. Physical parameters of the stimuli were measured using a spectrophotometer (Ocean Optics). Refresh rate of the CRT monitors (NEC Diamond Pro) were set at 60 Hz. We ran subset of experiments at a 200 Hz refresh on a specialized monitor (Iiyama HM204DT Japan) using a Nividia GeForce 8800 GT graphics card to check for effects of the refresh rate on fly behavioral responses in our assay. Results with a 200 Hz vertical refresh rate (presumably above the flicker fusion frequency for fly vision [5]) were not significantly different to 60 Hz refresh experiments (See Figure 2F). A standard green/black grating stimulus was used throughout, unless specified (green level 255, spatial frequency 0.016 cycles/degree, temporal frequency 3 Hz, velocity 130 degrees/s, Michelson contrast 1.0, based on a maximum of 255 and minimum of 0). Our standard dot stimulus, unless otherwise specified, was green level 255, 1500 dots on the screen, 100% motion coherence left or right, 10 s lifespan, 130 degrees/s, 13.8 degrees width. The visual parameters tested in each of the other experiments are described in the figure legends. See http://web.qbi.uq.edu.au/vanswinderen/Movie2.mpeg for the dot stimulus and http://web.qbi.uq.edu.au/vanswinderen/Movie3.mpeg for the combined dot/grating stimulus. To measure image regularity, movies were first decomposed into their individual frames and converted to black and white. The luminance levels along a horizontal line across the images were then Fourier transformed into frequency space using Matlab, and the power of the spatial frequencies was calculated for each image, yielding an average spectrum. The maximum power of each movie was compared with the Visual Response values for wild type and *dunce* ^1^, in a meta-analysis of the strains' performance in all of our combined movies (Figure 6D).

### Data Analysis and Statistics

Following an experiment (typically 8 mazes of ∼25 flies each), a Visual Response (VR) was automatically calculated (in Matlab, from Trikinetics counts) as a weighted average of flies in each terminal position of the maze (VR  =  (# flies in tube N)*N/(total # flies), where N = −4 to+4, or the location of the tube endings) [11]. A positive VR indicates that flies on average were turning in the direction of image motion displayed on the screen. The VR data was tested for normality using the Lilliefors test and a significant VR response was when the values were significantly different from zero, which was determined by *t*-test (or *U*-test for non-parametric data) against zero and set at *P*<0.01 for the psychophysics experiments and *P*<0.05 for other experiments. All data points are plotted as means ± standard error of the mean (s.e.m.). When images were presented in competition with one another (e.g., dots versus gratings), the grating direction was set as the baseline positive direction. Comparisons between strains for specific stimuli were done by *t*-test (or *U*-test for non-parametric data) and set at *P*<0.01 for significance. Comparisons between responses to different visual parameters were also made using one-way ANOVA, coorected for multiple comparisons, with *P*<0.01 set for significance. Pairwise linear correlations were also performed between the visual parameters and the VR values and for VR values between strains, and significant correlations set at *P*<0.05.
